# Fall prevention and vitamin D in the elderly: an overview of the key role of the non-bone effects

**DOI:** 10.1186/1743-0003-7-50

**Published:** 2010-10-11

**Authors:** Cedric Annweiler, Manuel Montero-Odasso, Anne M Schott, Gilles Berrut, Bruno Fantino, Olivier Beauchet

**Affiliations:** 1 Department of Internal Medicine and Geriatrics, Angers University Hospital; Angers University Memory Center; UPRES EA 2646, University of Angers, UNAM, Angers, France; 2 Department of Medicine, Division of Geriatric Medicine, University of Western Ontario, London, Ontario, Canada; 3 Department IMER, Lyon University Hospital; EA 4129, RECIF, University of Lyon; Inserm, U831, Lyon, France; 4 Department of Geriatrics, Nantes University Hospital; University of Nantes, UNAM, Nantes, France

## Abstract

Preventing falls and fall-related fractures in the elderly is an objective yet to be reached. There is increasing evidence that a supplementation of vitamin D and/or of calcium may reduce the fall and fracture rates. A vitamin D-calcium supplement appears to have a high potential due to its simple application and its low cost. However, published studies have shown conflicting results as some studies failed to show any effect, while others reported a significant decrease of falls and fractures. Through a 15-year literature overview, and after a brief reminder on mechanism of falls in older adults, we reported evidences for a vitamin D action on postural adaptations - i.e., muscles and central nervous system - which may explain the decreased fall and bone fracture rates and we underlined the reasons for differences and controversies between published data. Vitamin D supplementation should thus be integrated into primary and secondary fall prevention strategies in older adults.

## Introduction

Falls in the elderly are a public-health problem due to their high prevalence of 30% among subjects aged 65 and over, and their adverse outcomes [1-3]. In particular, fall-related fractures are associated with excess morbidity and mortality, and substantial financial cost [1-3]. In order to delay the occurrence of falls for as long as possible and to reduce its individual and public health impact, effective preventive interventions and strategies must be identified.

Falls can be prevented, as their incidence could be reduced by 18% by application of interventions in elderly community-dwelling subjects and by 25% in hospitalized subjects [1,2,4], regardless of the type of intervention. The intervention efficacy depends on two main principles: an interdisciplinary approach of health care professionals and a multifactorial approach in which regular physical activity has a key role [5,6]. However, application of this kind of intervention encounters two main problems. The first is the need for a network approach and the second is the poor compliance of elderly people in the proposed physical activity, regardless of its nature [7]. This last aspect is too frequently underestimated, but is central for the efficacy of any intervention designed to prevent falls. For example, Crombie et al. [7] showed that the main reason limiting the participation of elderly subjects in physical activity was their lack of interest in physical activity. In view of these two difficulties, together with the high financial cost of setting up population-based intervention measures, it is unlikely that the currently proposed fall prevention interventions and strategies will be easy to develop in the future.

Data accumulated since the original publication by Chapuy et al. [8] on the effects of vitamin D supplementation showed, despite several negative results [9-15], a reduction of the fall and bone fracture rates. As a consequence, a vitamin D-calcium supplementation, in contrast with the currently proposed fall prevention strategies, appears to have a high potential efficacy on fall and fracture reduction [16-23] due to its simple application and low cost.

## Increased fall risk in elderly individuals

According to the World Health Organization, a fall is defined as the action of finding oneself involuntarily on the ground. The prevalence of falls in the elderly is high and strongly correlated with age, increasing from 30% in subjects over the age of 65 to 50% in subjects over the age of 80 [1-3]. Falls represent the commonest accident of daily living and are the leading cause of accidental death in the elderly [1-3,24]. The severity of the fall is determined by its consequences including non-vertebral fractures, which essentially depend on the fall mechanism resulting in a variable force of impact on the ground [1,4,25-30].

Human is a biped and, thus, one of his characteristics is that the body's center of gravity is approximately located in the pelvis, i.e. perched high on a narrow support base [31]. To meet the demands of gravity, the body's equilibrium in the upright position is ensured when the center of gravity exerts a reaction force to the ground equal and opposite to the force of gravity in the vertical plane situated in the middle of the support base [31]. Balance can be disturbed by two types of events [31-35]. The first are "internal" disturbances, i.e. induced by the subject because expected, and for which anticipated postural adjustments (APA) precede the focal movement in order to counteract its destabilizing effects [31,33]. The second are unexpected, so-called "external" events derived from the environment [33]. Postural reactions triggered by this type of stimulus, designed to maintain balance, are rapid and automatic in healthy subjects [34]. It is classical to distinguish the ankle strategy, observed during slow and low-amplitude posterior translations of the weight bearing surface inducing anterior sway of the body [34,35]. The second strategy is the so-called hip strategy, which is used during rapid posterior or large-amplitude translations [35]. Selection of these strategies depends, apart from the nature of the disturbance, on the subject's state of attention and previous experience [31,36].

Maintenance of posture and balance during motor activities thus involves the reception and integration of multiple sensory afferents which inform the central nervous system (CNS) [1,37]. Reception and processing of all sensory information are ensured by the CNS, which responds by inducing a series of muscle contractions resulting in a series of coordinated movements, corresponding to adapted complex motor behavior [38,39]. For example, the walking process is related to the numerous demands that an individual needs to process simultaneously when walking: firstly, propulsion of the body in the horizontal plane via postural constraints including slowing body segments that have a high kinetic energy and may create a dynamic imbalance; secondly, maintenance of a stable equilibrium by ensuring a coordination between posture and movement; and thirdly, adaptation at any moment of time to environmental constraints [28,32].

It has been suggested that the specificity of the mechanism of falls in the elderly, particularly the impairment of postural reactions - either altered or delayed - could partly explain the higher incidence of hip fractures compared to wrist fractures after the age of 75 [29,30,36]. The inappropriate nature of postural reactions, either responsible for or occurring during a fall, is due to an abnormality of processing of musculoskeletal mechanisms and of sensorimotor information in the CNS. The central question is to determine whether the age-related alteration of the postural adaptation abilities - through the central nervous integration and peripheral muscular effectors - could be related to vitamin D and calcium status (normal or insufficiency) and/or the use of replacement therapy in this age-group. The literature provides arguments in favor of such an association.

## Vitamin D and postural adaptations

### Metabolism and mechanism of action of vitamin D

Vitamin D is a fat-soluble vitamin synthesized from a cholesterol derivative [18,38]. It exists in two forms: vitamin D_2 _or ergocalciferol, which is produced by irradiation of ergosterol (provitamin D provided by the diet) by the action of ultraviolet (UV) radiation in the skin, and vitamin D_3 _or cholecalciferol provided directly by foods or produced by the action of UV from cholesterol after transformation into 7-dehydrocholesterol [38,39]. In the liver, cholecalciferol is transformed into calcifediol or 25(OH)D, which enters the blood circulation, then, in the renal tubular cells, calcifediol is hydroxylated into calcitriol or 1,25-dihydroxyvitamin D (1,25(OH)D) which is the active form of vitamin D [38,40].

Vitamin D is a steroid hormone [41] because of its mechanism of action which is exerted either directly on membrane receptors affecting extracellular and intracellular concentrations of Ca^++ ^via calcium channels and which define the nongenomic action, or by binding to nuclear receptors, which determines the genomic action [40,42,43]. In this second case, the vitamin D/receptor complex formed induces the synthesis of messenger ribonucleic acid (mRNA) which codes for a protein, Calcium Binding Protein (CaBP), responsible for the biological effect [43,44]. This type of action takes longer to be effective than the nongenomic action [42].

For a long time, the main role of vitamin D was considered to be the regulation of calcium and phosphate metabolism [16], in which bone was the main target organ and its action was considered to be limited to cell turnover by increasing the life span of osteoblasts by an anti-apoptosis effect [44]. However, recent data suggest that muscles and the nervous system are also target organs of vitamin D.

## Vitamin D and muscles

### Clinical evidence

First of all, several lines of clinical evidence suggest the existence of a link between vitamin D and muscle function. Cases of myopathy have been described in severe vitamin D insufficiency, responsible for rickets in children and osteomalacia in adults [45-49]. These severe forms of vitamin D insufficiency cause severe muscle weakness, usually proximal and involving the lower limbs [48,49]. Apart from these extreme cases, vitamin-insufficient myopathies are generally underdiagnosed due to the progressive and continuous onset of nonspecific clinical signs such as muscle pain, paraesthesiae or arthralgia, which are initially suggestive of a diagnosis of inflammatory rheumatic disease [49]. Clinical signs may also not necessarily be related to muscle lesions as, in a series of 30 cases, Skaria et al. [47] showed that, although clinical signs were present in more than 95% of cases, only 30% of muscular biopsies revealed histological signs of vitamin D insufficiency-related myopathy. In case of severe vitamin D deficiency with osteomalacia, these signs are associated with widening of interfibrillary spaces, fatty infiltration, fibrosis and the presence of glycogen granules, with no signs of inflammatory reaction [47,48]. It has also been shown predominantly type II muscle fibres atrophy [50], while vitamin D repletion instead leads to an increase in relative fibres composition and in fibres area of type IIa muscle fibres [51,52]. It remains yet unclear if the increase in type II muscle fibre number is caused by new formation of type II fibres or a transition of already existing fibres from type I to type II [53].

### Molecular mechanisms

Second, experimentation revealed that the genomic pathway of vitamin D action in muscle involves activation of 1,25(OH)D nuclear receptors that triggers the production of messenger RNA and the synthesis of proteins responsible for multiple phenomena such as calcium influx into the cell, membrane phosphate transport, phospholipids metabolism, and muscle fibre proliferation and differentiation [38-40,44]. This genomic pathway of action of vitamin D also influences the polymorphism of VDR responsible for the nongenomic pathway of action [43]. This nongenomic pathway has a complementary action to that of the genomic pathway either by activating a second messenger in the cell - cyclic AMP and/or diacylglycerol and/or inositol triphosphate and/or arachidonic acid - or by activating protein kinase C and the release of calcium into the cytosol [54,55]. This effect is responsible for the active transportation of calcium into sarcoplasmatic reticulum by Ca-ATPase increasing the calcium pool which is necessary for the successive attachments and detachments of myofilaments leading to sarcomeric shortening responsible for muscular contraction [56]. Vitamin D therefore participates in the good functional equilibrium of fast-twitch type II muscle fibres, thereby preserving high muscle contraction speed and muscle power [38-43,56].

### Observation: mixed results

In epidemiological studies, the relationship between vitamin D and muscle function remains more controversial, as it has been inconsistently described [45]. For instance, Bischoff-Ferrari et al. [57] observed, in a population of 319 community-dwelling subjects with a mean age of 75.9 years, that a 25(OH)D rate ≤ 12 ng/mL was significantly correlated with decreased leg extension strength, with a less intense effect in women compared to men. However, after adjustment for gender, age, body mass index and serum parathormone, this correlation was no longer significant [57]. Annweiler et al. obtained similar results amongst community-dwelling older women aged 75 and older from the EPIDOS cohort [58,59]. They found a significant association of low serum vitamin D with low quadriceps strength [58] and handgrip strength [59] in the unadjusted model, but these associations were not significant anymore after adjustment for age, body mass index, number of chronic diseases, practice of regular physical activity, serum calcium concentration, creatinine clearance, and hyperparathyroidism [58,59]. In contrast, Mowe et al. [60], in a population of hospitalized subjects (n = 246) and subjects living at home (n = 103) between the ages of 70 and 91 years, showed that, regardless of the group considered, the serum 25(OH)D concentration was correlated with the grip strength of the non-dominant hand, difficulty climbing stairs, and regular physical activity. Finally, Kuczynski and Ostrowka [61] reported indirect evidence that low bone mineral density in osteoporotic elderly women presenting vitamin D insufficiency was associated with increased postural sway in the mediolateral plane.

### Intervention: mixed results

Like observation studies, intervention studies have demonstrated discordant results concerning the effects of vitamin D supplementation on muscle function [21,45]. In a literature review published in 2003 and based on 33 clinical trials and a total population of 2,496 elderly subjects, only 3 trials showed a significant improvement of muscle strength and/or physical performance [21]. In these 3 trials, the vitamin D supplement was associated with calcium. However, when trials presenting methodological bias were excluded, only one trial demonstrated a significant improvement. More recently, Annweiler et al. [45] conducted a systematic review which confirmed that the relationship between vitamin D and muscle function was controversial in clinical trials as some studies found a significant vitamin D-related improvement in physical performance, while others failed to show any effect of supplementation. These divergences highlighted the fact that the effects of vitamin D supplementation were directly correlated with the initial severity of vitamin D insufficiency [49]. Vitamin D supplementation has also been reported to act significantly and specifically on so-called antigravity muscles [61]. This action of vitamin D on muscle has been shown to play a role in maintenance of postural equilibrium. Dhesi et al. [62] reported that an intramuscular injection of 600,000 IU ergocalciferol in 70 subjects with a mean age of 76.6 ± 6.1 years, a history of falls and a 25(OH)D concentration ≤ 12 ng/mL versus an intramuscular placebo injection in a group of 69 matched subjects, significantly reduced postural sway. In this study, a 3% increase of the amplitude of sway was observed in the placebo group, while the amplitude of sway decreased by 13% in the intervention group. However, this study did not demonstrate any effect on muscle strength. Binder et al. [63] demonstrated that vitamin D and calcium supplementation significantly improved postural equilibrium tests.

The failure to demonstrate any positive effect of vitamin D on muscle performance could also be related to the duration of follow-up after starting treatment, which did not exceed 6 months in the majority of cases [21,45], whereas the effect of vitamin D may be observed later. For instance, in the case of biopsy-documented myopathy, vitamin D supplementation restores muscle after a period of 6 to 12 months [46-49]. Furthermore, the latest publications of experimental research on vitamin D receptors (VDR) suggest the existence of responders and non-responders to vitamin D. For example, Wang et al. [64] showed that a given VDR genotype corresponds to a given intensity of muscle strength, as these authors observed, in a population of 109 young women, that the AA homozygous genotype of ApaI VDR was associated with lower muscle strength than the aa or Aa homozygous genotypes. Similarly, the bb homozygous genotype of BsmI VDR was associated with lower muscle strength than the BB or Bb homozygous genotype. On the other hand, no difference was demonstrated between the various TaqI VDR genotypes [64].

Furthermore, Stein et al. [65] have suggested that the muscle effect of vitamin D insufficiency could be due to parathormone and not to a direct action of vitamin D on muscle. Vitamin D insufficiency triggers a series of reactions, including elevation of serum parathormone concentrations [38-42]. Serum parathormone appears to be an indirect tissue marker of vitamin D insufficiency that is more specific than the serum vitamin D concentration itself [65], as serum 25(OH)D has been demonstrated to be poorly correlated with the muscular tissue response [40]. Furthermore parathormone has a muscle action that is independent of vitamin D [22]. More specifically, studies in rodents have demonstrated that parathormone induces muscle catabolism [66], reductions in calcium transport (i.e., Ca-ATPase activity) and impairment of energy availability (with reduction in intracellular phosphate and mitochondrial oxygen consumption) and metabolism (including reduction in creatinine phosphokinase and oxidation of long-chain fatty acids) in skeletal muscles [67]. This relationship between serum parathormone and muscles has been known for a long time in patients with primary hyperparathyroidism, whose clinical features comprise fatigue and muscle weakness [40,42]. These symptoms improve after parathyroidectomy [68]. Furthermore, parathormone has been shown to predict falls [65] and muscle strength independent of 25(OH)D [69]. The specific roles of vitamin D and parathormone on muscle are thus not fully elucidated [68].

Given the divergence in published results, it appears that vitamin D could affect neuromuscular function and fall risk in a way which does not involve only the muscle but also the CNS.

## Vitamin D and nervous system

### Molecular mechanisms

As in muscle, vitamin D acts according to genomic and nongenomic pathways [39-42]. VDR have been demonstrated in some parts of the brain, especially in the hippocampus, hypothalamus, and limbic system but also in cortical, subcortical and spinal motor zones [70-78]. At the cellular level, these receptors are present on neurons and glial cells [40-74].

Experimentally, in animals, vitamin D is involved in neurophysiology and regulates the metabolism of neurotransmitters including dopamine, acetylcholine, serotonin and gamma aminobutyric acid [70,78], and the synthesis of certain growth factors such as Nerve Growth Factor (NGF) or Glial cell line-derived neurotrophic factor (GDNF) [70-77]. Vitamin D is also involved in the development and maturation of rodents brain [70,71,75]. In addition to this central action, vitamin D also acts on the peripheral nervous system, as a reduction of nerve conduction velocity has been reported in the case of severe vitamin D insufficiency [47].

Vitamin D is also involved in neuroprotection through immunomodulating, anti-ischemic and anti-oxidative properties. Indeed, trophic induction plays a neuroprotective role in cerebral ischemia [79], as well as an anti-neurodegenerative role for dopaminergic cells in models of Parkinson's disease [80]. Moreover, it seems that vitamin D plays a part in the cerebral processes of detoxification by interacting with reactive oxygen and nitrogen species in rat brain and by regulating the activity of γ-glutamyl transpeptidase [81], a key enzyme in the antioxydative metabolism of glutathione. Concentrations around 0.1 to 100 nanomoles of 1,25(OH)D thus ensure an efficient protection of neurons against the direct effects of superoxyde ions and hydrogene peroxyde [80]. Finally, VDR-dependent immunosuppressive effects, including increased concentrations of inflammatory cytokines, macrophages, polynuclears, as well as their sensitization to apoptotic signals, were described in the CNS [70]. For illustration, in a model of mice with experimental allergic encephalitis, 1,25(OH)D inhibited autoimmune neurological processes [82,83].

Vitamin D could also be vasculoprotective since vitamin D insufficiency has been associated with incident cerebrovascular disease [84]. For instance, atherosclerosis is a systemic inflammatory disease related to vitamin D insufficiency [85]. C-Reactive Protein is a marker of inflammation and atherosclerosis regulated by Interleukin-6 (IL-6) and Tumor Necrosis Factor-α (TNF-α) [86], which secretions dose-dependently decreased in presence of vitamin D [87]. Furthermore, vitamin D insufficiency could be a contributing factor to hypertension - a major determinant of the development of cerebrovascular diseases - by the suppression of the renin-angiotensin system expression in the juxtaglomerular apparatus [88] and by an action on the arterial wall compliance [88,89].

All together, these properties could stabilize the neurophysiologic function and explain why the lack of functional VDR in the brain of VDR-knockout transgenic mice models was responsible for behavioral disorders due not only to an increased level of stress but also to severe motor disorders [73,78,90-92]. For instance, the suppression of functional cerebral VDR in transgenic mice induced a decreased swimming capacity with fewer swimming movements, suggesting the essential role of vitamin D in motor control [90].

### Observation

Some clinical data in humans appear to support the hypothesis of a favorable action of vitamin D on cognitive function, especially attention, as Yaffe et al. [93] observed, in a population of 8,333 women over the age of 65, that cognitive performance on frontal and attentional tests were lower in women with a low BMD or vertebral fractures, establishing a link between post-menopausal osteoporosis - related to vitamin D insufficiency - and cognitive decline. Although the hypothesis of a simple temporal relationship is possible in this study, the hypothesis of an action of vitamin D on cognitive function is highly likely [94]. In particular, epidemiological studies revealed lower serum 25(OH)D concentrations in subjects with Alzheimer disease than in healthy subjects [95,96]. In addition, emerging analytical studies have brought new evidence [94]. For instance, Wilkins et al. [97] found a significant positive association between the serum 25(OH)D levels and the scores at the Clinical Dementia Rating and at the Short Blessed Test in 80 older subjects aged 65 and over, living at home (40 subjects with AD and 40 non-demented subjects). Additionally, Przybelski et al. [98] and Oudshorn et al. [99] highlighted an association with the Mini Mental Status Examination (MMSE) score. Similarly, Llewellyn et al. demonstrated among 1,766 non-demented subjects or with Mild Cognitive Impairment aged 78 years on average that the lowest 25(OH)D concentrations, the highest risk of pathological Abbreviated Mental Test score [100]. In line with this, Annweiler et al. showed a 2-fold risk of global cognitive impairment (Pfeiffer's Short Portable Mental State Questionnaire) among 752 older women (mean age 82 years) [101]. Finally, Buell et al. [102] showed among 318 participants (mean age 73.5 years, 72.6% women) that 25(OH)D insufficiency was associated with more than twice the odds of all-cause dementia and of Alzheimer disease. In contrast, two studies found no significant association [103,104]. First, Jorde et al. have unsuccessfully explored the linear association of 25(OH)D with 6 specific cognitive functions (working memory, episodic memory, speed of information processing, language, executive functions and intelligence) in 148 older subjects with hyperparathyroidism (mean age 62 years, 46% women) [103]. Second, McGrath et al. found no significant positive logistic association between the quintiles of serum 25(OH)D concentrations and several specific cognitive tasks among 4,747 adults between 20 and 59 years (Symbol-digit Substitution Coding Speed: attention and episodic memory; Serial Digit Learning Trials To Criterion: working memory) [104].

From a prospective perspective, Slinin et al. [105] highlighted a trend for an independent association between lower 25(OH)D levels and odds of cognitive decline by Modified Mini Mental State score among 1,604 men enrolled in the Osteoporotic Fractures in Men Study and followed for an average of 4.6 years. Additionally, Llewellyn et al. [106] showed that low 25(OH)D levels were associated with substantial decline in MMSE score among 858 adults aged ≥65 years studied over a 6-year period.

Literature review shows that the choice of confounders is essential and could explain the divergences in results. Analyses should thus take into account a list of covariates such as depression or serum parathormone concentrations.

First, depressive mood is associated with both cognition and vitamin D. Indeed, depression by itself can mimic dementia - when people are depressed, they can have difficulty concentrating, which leads to forgetfulness - or is often part of dementia, or may cause by itself executive dysfunction [107]. Additionally, a relationship between vitamin D deficiency and anxio-depressive disorders is likely since low serum 25(OH)D concentrations are closely associated with active mood disorders [70] and have been proposed as the missing link between seasonal changes in photoperiod and seasonal mood swings [70]. In line with this, one clinical trial supported the efficacy of vitamin D supplementation on mood disorders [108]. Finally, accounting for depression is of primary importance while exploring the involvement of vitamin D-related cognitive functioning in locomotor function as depressed people are usually less active and loose muscle mass as well as sensorimotor performance [70].

Second, vitamin D belongs a complex biological system, and its insufficiency causes an elevation of serum parathormone [109]. Patients with primary hyperparathyroidism usually exhibit cognitive disorders [109,110], that could be reversed after parathyroidectomy [110]. Moreover, in the Helsinki Ageing Study, high parathormone concentrations indicated an independent 2-fold risk for a five-year cognitive decline [111]. The systemic microvascular disease involving cerebral vasculature together with hypercalcemia have been proposed to result in disruption of the blood brain barrier and accumulation of calcium deposits in brain tissue, leading to cognitive impairment [111]. In vitro studies have also shown that parathormone increases intracellular calcium concentration and causes cell deterioration in the rodent hippocampal neurons [112]. Furthermore, individual differences in the cell membrane ability to resist calcium influx have been hypothesized to cause the well-known but poorly understood variability of clinical symptoms in patients with hyperparathyroidism [111].

Anyway and to the best of our knowledge, the association of hypovitaminosis D with global cognitive impairment persist after adjustment for these both covariables.

This association of vitamin D with global composite cognitive scores has been recently explained by executive function and processing speed impairments [106,113]. Amongst 1,080 subjects (mean age 75 years, 76% women) free of neuropsychiatric disorders (epilepsy, schizophrenia, bipolar disorder, mental retardation, brain tumors, Human Immunodeficiency Virus), Buell et al. found a significant positive linear association between serum 25(OH)D concentrations and scores in tests exploring executive functions (Trail Making Test: flexibility) and speed of information processing (Digit Symbol Coding) [113]. In addition, Llewellyn et al. [106] found a substantial decline on Trail-Making Test B among 858 adults 65 years or older enrolled in the InCHIANTI study and followed for an average of 5.2 years. Executive functions include all heterogeneous cognitive processes required in the regulation of cognitive activity during the treatment of complex and/or new and/or conflictual tasks [114]. These frontal and attention functions are precisely those which enable us to adapt our behaviors - such as walking - to expected or unforeseen situations of daily living. They are therefore of prime importance for determining posture, navigation abilities and locomotor performance. For instance, they have direct impact on selection of postural control strategies when older adults encounter specific temporal and environmental constraints which could place them at risk for falls [114-116].

## Intervention

Vitamin D appears to stabilize postural equilibrium in the elderly via an improvement of attention capacities independently of any muscular action, as Dhesi et al. demonstrated that vitamin D supplementation in elderly fallers significantly decreased reaction times to stimuli and improved postural equilibrium independently on any effect on muscle [69]. The same authors have already demonstrated this effect on the CNS in a group of elderly fallers, by showing that low serum vitamin D concentration was independently associated with high amplitude of postural sway and vice versa [62]. In line with this, vitamin D has been linked to walking speed and acceleration capacity [117], and vitamin D supplementation improved walking performance [118] by mechanisms involving not only muscles but also nervous system [117].

From a cognitive perspective, it has been demonstrated that, in elderly rats, vitamin D reduced inflammatory disorders and hippocampal degenerative processes, and was also responsible for decreased levels of the biological markers of ageing [70]. In humans, Annweiler et al. [119] showed a significant association between weekly vitamin D dietary intakes and global cognitive function, and found that inadequate weekly vitamin D dietary intakes were associated with cognitive impairment among 5,596 community-dwelling healthy older women aged 80.4 years on average. However, to the best of our knowledge, no randomized controlled trial on the efficacy of vitamin D on cognition has been conducted to date.

Based on these elements, the hypothesis that vitamin D influences the occurrence and mechanism of the fall and its consequences due to its action on postural balance system - i.e., CNS and muscles - would then be feasible.

## Evidence of the effectiveness of vitamin D on falls and bone fractures

### Epidemiology of vitamin D-related falls

From an epidemiological point of view, vitamin D insufficiency is very frequent in the elderly and is dependent on the presence or absence of a history of falls [120,121]. The prevalence of vitamin D insufficiency is estimated between 40% and 50% in non-fallers over the age of 65 and up to 70% in fallers [65,120,121]. It has also been demonstrated in institutionalized elderly that fallers had lower serum vitamin D concentrations than non-fallers [121].

In addition, the majority of data published over the last 15 years demonstrated the existence of a significant effect of vitamin D and/or calcium supplementation on fall reduction [16,17]. It has indeed been shown that vitamin D supplementation (800 IU/day) either alone or in combination with calcium (500-1200 mg/day) allows a very marked reduction in the number of falls in the same individual but also in the number of fallers, with a reduction of up to 50% [16-18]. A 2004 meta-analysis confirmed that simple vitamin D supplementation, regardless of its type but at a dose of 800 IU/day, allowed a mean reduction of the fall rate by 22%, with a maximum effect of 53% when combined with oral calcium [16]. This meta-analysis also showed that the number of subjects needed to treat to prevent one fall was 15. Furthermore, the most recent meta-analysis by Bischoff-Ferrari et al. [17] demonstrated that vitamin D supplementation of at least 700UI per day might reduce the risk of falls amongst older adults by 19%. In addition, a minimum serum vitamin D concentration of 60 nmol/L could result in a 23% fall reduction, whereas lower concentrations had no effect on the number of falls [17].

### Epidemiology of vitamin D-related fractures

In addition to vitamin D-related phosphocalcic regulation, the vitamin D-related fall rate reduction induces a fracture rate reduction. A 2005 meta-analysis on the antifracture effect of vitamin D supplementation based on 12 clinical trials combining a total of 19,114 women over the age of 60 and living at home showed a significant reduction of the relative risk of hip fracture by 26% and other non-vertebral fractures by 23% [22]. This antifracture effect was only observed for a vitamin D supplementation of 700 to 800 IU per day. A similar result was observed in frail institutionalized elderly subjects [65]. In contrast, the Cochrane Systematic review concluded that there was no reduction in fracture rate related to vitamin D supplementation alone [18], while combined calcium and vitamin D supplementation reduced significantly the incidence of fractures in older adults living in institutionalized care facilities [18], which was confirmed by two 2007 meta-analyses [122,123]. In line with this, a third 2007 meta-analysis concluded that calcium with or without vitamin D may reduce the total fracture risk by 12% [41]. Finally, Bischoff-Ferrari et al. [23] most recently demonstrated in a 2009 meta-analysis of high-quality double-blinded randomized clinical trials - including 42279 adults aged 65 and older - the protective action of oral supplemental vitamin D against nonvertebral fractures with a dose dependant effect. This prevention was effective whether in community-dwelling or institutionalized older individuals, and was interestingly independent of additional calcium supplementation [23].

### Incongruous data

However, some negative results appear to contradict these previous findings, as they failed to demonstrate any significant fall or fracture reduction [9-15] (Table 1). These mixed results could be due to potential confounders. Firstly, the vitamin status appears to be essential, as vitamin D insufficiency, defined according to a serum cut-off value ranging between 10 and 30 ng/mL of 25(OH)D, is more often associated with a significant effect [8]. Secondly, the daily dose of vitamin D is decisive and must be at least 800 IU per day. For instance, in Jackson et al.'s study [13], the dosage was only half that recommended to obtain an effect on the risk of falls. Thirdly, subjects must comply with treatment. In Jackson et al.'s study [13], negative results were obtained by intention-to-treat analysis, but in this study, only 59% of women presented good compliance with vitamin D and calcium treatment, defined by the authors as taking 80% or more of the prescribed treatment. When the analysis was limited to women with good compliance with treatment, the effect on reduction of hip fractures was significant with a 29% reduction of the fracture rate. Calcium and vitamin D supplementation was also associated with a 26% reduction of the fall rate for women with no history of falls. Fourthly, the initial health status of elderly subjects seems also decisive, as it directly influences the risk of falls and complications [124]. As an example, in Cochrane Systematic review, the effect of combined vitamin D and calcium on fractures was solely shown in institutionalized subjects [18]. Ageing, either physiological or pathological, is a process which modifies the individual's health status. At the population level, it results in the formation of a heterogeneous group in terms of health status [11,124-126] comprising a subgroup of high-risk subjects with an altered state of health due to multiple diseases, with functional limitations and impaired adaptation capacities and a high risk of falls [124-126]. The mixed conclusions could also depend on selection of studies for inclusion in the meta-analyses [16,17,21]. As an example, a negative study was excluded from the last meta-analysis because patients were "in an unstable health state" although it was not an initial exclusion criterion [17,127]. It should also be noted that several studies showed that vitamin D2 was less effective than vitamin D3 in humans [128-130]. In addition, the absence of effect of vitamin D supplementation on fractures could depend on the type of fracture considered [10-15]. Finally, fall was usually not the primary outcome in these studies and assessment of fall frequency was not optimal [10-15].

**Table 1:** Studies that failed to demonstrate any significant effect of vitamin D and/or calcium supplementation on fall and bone fracture rate reduction in the elderly

Study	Primary objective	Study plan	Population	Supplementation	Results
Latham et al. 2003 [9]	- I Prevention area and II area	- Multicenter, randomized, controlled trial with a factorial design	- N = 243	- Vitamin D:	- No reduction of the fall rate
	- Falls	- Mean follow-up: 6 months	- Mean age = 79.1 ± 6.9 years	Calciferol Dose: 300,000 IU Per os	
			- Women: 53%	- Compliance: 100%	
			- Subjects:		
			Frail hospitalized History of falls in previous year: 56% Mean 25(OH)D_3 _at entry = 17 ng/mL		
					
Smith HE et al. 2004 [10]	- Primary prevention	- Randomized, controlled, double-blind trial	- N = 9,440	- Vitamin D:	- No significant reduction of fracture risk (OR = 1.10; [0.94-1.29]
	- Non-vertebral fractures	- Mean follow-up: 3 years	- Age > 75 years	Ergocalciferol (D_2_) Dose/year: 300,000 IU IM	
			- Women living at home		
					
Porthouse J et al. 2005 [11]	- Secondary prevention	Randomized, controlled, open-label trial	- N = 3,314 (1,993 controls and 1,321 intervention)	- Vitamin D:	- No significant risk reduction:
	- Vertebral or long bone fracture	- Mean follow-up: 2 years	- Mean age: (76.5 ± 5.0 control and 77.0 ± 5.1 intervention)	Cholecalciferol (D_3_) Dose/day: 800 IU Per os	Fractures (OR = 1.01; [0.71-1.43] Falls (OR = 0.99 [0.81-1.20] at 6 months; OR = 0.98 [0.79-1.20] at 12 months
			- Women:	- Calcium: 1000 mg/day	
			Living at home With one or more risk factors for hip fracture History of falls: 34%	- Compliance: 63% at 12 months and 55% at 24 months	
					
Grant AM et al. 2005 [12]	- Secondary prevention	- Randomized, controlled, double-blind trial	- N = 5,292 (1,332 controls and 1,311 calcium, 1,343 vitamin D and 1,306 Vitamin D and calcium)	- Vitamin D:	- No significant risk reduction:
	- Vertebral or non-vertebral fractures	- Mean follow-up: 2 years	- Mean age	Cholecalciferol (D_3_) Dose/day: 800 IU Per os	Fractures (OR = 0.94; [0.81-1.09] for calcium) (OR = 1.02; [0.88-1.19] for vitamin D) (OR = 1.01; [0.75-1.36] for the Vitamin D and calcium combination)
			- Women:	- Calcium: 1000 mg/day	
			Living at home With one or more risk factors for hip fracture History of falls: 34%	- Compliance: 54.5% at 24 months	
					
Jackson RD et al. 2006 [13]	- I Prevention area and II area	- Randomized trial, controlled, double blinds	- N = 36,282 (18,106 controls and 18,176 intervention)	- Vitamin D:	- No reduction of the fracture risk (OR = 0, 96; [0.91-1.02]
	- Vertebral fracture or long bone	- Mean follow-up 7 years	- Mean age (62.4 ± 6.9 years control subjects and 62.4 ± 7.0 years for intervention subjects)	Cholecalciferol (D3) Dose/day: 400 IU Per os	No effect of serum vitamin D3 level
			- Women:	- Calcium: 1000 mg/day	
			Living at home In good health Post-menopausal osteoporosis History of falls: 39%	- Compliance: 63% at 3 years and 59% at 7 years	
					
Law M et al. 2006 [14]	- I Prevention area and II area	- Randomized trial, controlled, opened	- N = 3,717 (1,955 controls and 1,762 intervention)	- Vitamin D:	- No reduction of the rate of falls or the incidence of fractures.
	- Vertebral fracture and long bone, and fall	- Mean follow-up 10 months	- Mean age of 2 groups 85 years	Ergocalciferol (D2) Dose/3 months: 1,100 IU Per os	
			- 76% of women in each group		
			- Subjects:		
			> 60 years Institutionalized		
					
Lyons RA et al. 2007 [15]	- I Prevention area and II area	- Randomized trial, controlled, double blinds	- N = 3,440 (1,715 controls and 1,725 intervention)	- Vitamin D:	- No reduction of the incidence of fractures
	- Vertebral or non-vertebral fractures	- Mean follow-up 3 years	- Mean age (84 ± 7.4 years control subjects and 84 ± 7.6 years for intervention subjects)	Ergocalciferol (D2) Dose/4 months: 100,000 IU Per os	
			- 76% of women	- Compliance: 80% at 3 years	
			- Subjects:		
			Institutionalized		

## Conclusions

Falls in the elderly, as well as fall-related adverse outcomes such as low trauma bone fractures, are events that could be prevented. Epidemiological studies conducted over the past 15 years provide an increasing number of arguments in favor of an action of vitamin D on muscles and CNS. Vitamin D improves postural balance, propulsion and also executive functions and navigation abilities among older adults. Vitamin D supplementation thus not only determines gait performance, but also prevents the occurrence of falls and their complications among older adults. Mixed data regarding the absence of effect of vitamin D and calcium supplementation are mainly due to the fact that some confounders were not taken into account, but also to the baseline serum vitamin D concentration on initiation of treatment, as a low serum vitamin D concentration appears to be associated with better efficacy. The prescription of at least 800 IU of vitamin D daily in insufficient elderly subjects is a simple intervention that should be incorporated into new strategies for postural rehabilitation, primary and secondary fall prevention, strength training, integration of body schema, automation of gait and adaptation to the environment.

## Abbreviations

BMD: Bone mineral density; CNS: Central nervous system; APA: Anticipated postural adjustments; 25(OH)D: 25-hydroxyvitamin D; UV: Ultraviolet; 1,25(OH)D: 1,25-dihydroxyvitamin D; mRNA: Messenger ribonucleic acid; CaBP: Calcium Binding Protein; OR: Odds ratio; VDR: Vitamin D receptor; NGF: Nerve Growth Factor; GDNF: Glial cell line-derived neurotrophic factor; MMSE: Mini Mental Status Examination.

## Competing interests

CA serves as a consultant for Ipsen Pharma company. He has no relevant financial interest in this manuscript. MMO reports no conflict of interest. He has no relevant financial interest in this manuscript. AMS serves as a consultant for Ipsen Pharma company. She has no relevant financial interest in this manuscript. GB reports no conflict of interest. He has no relevant financial interest in this manuscript. BF reports no conflict of interest. He has no relevant financial interest in this manuscript. OB serves as a consultant for Ipsen Pharma company. He has no relevant financial interest in this manuscript.

## Authors' contributions

CA has full access to the data in the study and takes responsibility for the integrity of the data and the accuracy of the data analyses. Study concept and design: CA and OB. Acquisition of data: CA and OB. Analysis and interpretation of data: CA, OB, MMO, AMS, and BF. Drafting of the manuscript: CA and OB. Critical revision of the manuscript for important intellectual content: MMO, AMS, GB, and BF. Obtained funding: not applicable. Administrative, technical, or material support: CA and OB. Study supervision: OB. All authors read and approved the final manuscript.
